# Non-selective enhanced recovery pathway in primary hip and knee arthroplasty: a propensity score matched analysis on safety and efficacy

**DOI:** 10.1186/s42836-026-00367-w

**Published:** 2026-02-09

**Authors:** Amr Selim, Deepak Menon, Eleanor Rouse, Rebecca Warren, Dan Redfern, Samantha Davies, Niall Graham, Geraint Thomas

**Affiliations:** 1 Robert Jones & Agnes Hunt Orthopaedic Hospital, Oswestry, SY10 7AG UK; 2 School of Medicine, Keele University, Staffordshire, SY10 7AG UK

**Keywords:** Enhanced Recovery, Hip Arthroplasty, Knee Arthroplasty, Non-Selective

## Abstract

**Background:**

Enhanced Recovery After Surgery (ERAS) was introduced in hip and knee arthroplasty to expedite recovery, shorten inpatient stay, and reduce costs. This study aims to investigate the safety and efficacy of implementing a universal standardized non-selective ERAS service for all patients admitted for primary hip and knee arthroplasty in a single high-volume tertiary orthopaedic centre.

**Methods:**

All patients who underwent primary hip or knee arthroplasty under ERAS from April 2023 to March 2024 were compared with a matched cohort between January 2018 and December 2019. Patients were matched at a 2:1 ratio based on procedure, age, sex, ASA grade, and BMI (ERAS = 1811, Standard Care = 3549 patients). Outcomes included Length of Stay (LOS), 30-day readmission, overall infection, superficial infection, deep infection, 30- and 90-day mortality rates.

**Results:**

The median LOS was 1 day (IQR 1–2) in the ERAS group versus 3 days (IQR 2–4) in the Standard Care group (W = 5,415,769, *P* < 0.001). Rates of 30-day readmission (1.7% vs. 2.1%), overall infection (0.66% vs. 1.15%), deep infection (0.39% vs. 0.68%), superficial infection (0.28% vs. 0.48%), 30-day mortality (0.11% vs. 0.20%), and 90-day mortality (0.22% vs. 0.37%) were all higher in the Standard Care group. However, these differences were not statistically significant, with *P*-values of 0.41, 0.11, 0.26, 0.38, 0.70, and 0.52, respectively. The estimated cost reduction per patient with the ERAS pathway, considering only the difference in LOS, is £718.60(95%CI £602.56 to £832.64). The subgroup analysis for patients ≥ 80 revealed a statistically significant difference in LOS, which was more pronounced with a median difference of 3 days (5 days in standard care versus 2 days in ERAS, *P* < 0.001).

**Conclusion:**

Non-selective ERAS was safe and effective in reducing LOS for patients undergoing primary THA and TKA across all age groups and varying comorbidity statuses. Although perioperative morbidity and mortality were less in ERAS, these changes did not reach statistical significance.

## Introduction

Total Hip and Knee Arthroplasties (THA & TKA) have seen significant growth over the last decade [1]. A report published in 2014 stated that by 2030, THA procedures in the US are expected to increase by 71%, reaching 635,000 annually, while TKA procedures are projected to grow by 85%, reaching 1.26 million annually [2]. With this exponential growth, improving patient outcomes and cost-effectiveness has become a priority [3, 4]. One key factor in achieving these goals is expediting recovery and shortening patient length of stay [4].

Enhanced Recovery After Surgery (ERAS) protocols for hip and knee arthroplasty have been advocated over the last decade as effective measures to improve time to recovery, reduce length of stay, and potentially reduce complications and mortality [4, 5]. These pathways focus primarily on perioperative patient optimization [6, 7]. The ERAS group has provided several recommendations to ensure successful implementation, including preoperative education, smoking and alcohol cessation, intraoperative local anaesthetic infiltration, specific anaesthetic protocols, and setting discharge criteria with milestones to ensure patient safety [8]. Notably, while these pathways change perioperative management significantly, the surgical procedure itself remains largely unchanged [9].

However, most ERAS pathways are selective, typically including younger or healthier patients [10–12]. A recent systematic review of 7,789 patients reported that ERAS effectively reduced length of stay, with a greater impact in healthier patients [10].

There has been little emphasis on non-selective ERAS protocols that include all patients regardless of comorbidity status or age. Such non-selective protocols could be more applicable to the broader healthcare system rather than certain specialized units.

This study aims to report the clinical impact of implementing a non-selective, standardized ERAS service for all patients admitted for primary total hip and knee arthroplasty. An assessment of compliance with critical aspects of the ERAS protocol was undertaken, and the safety and efficacy of this non-selective ERAS pathway were assessed.

## Methods

**Fig. 1:**
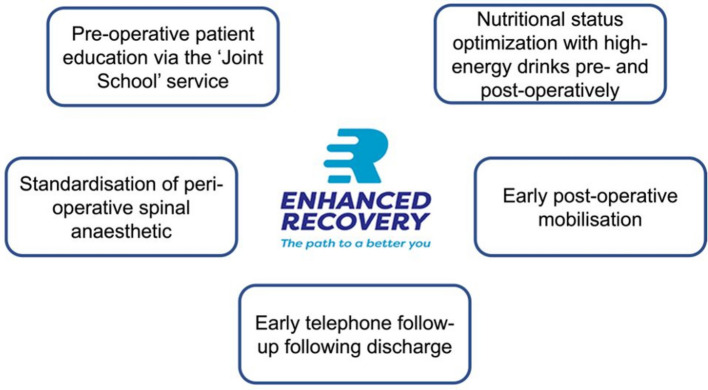
Components of the enhanced recovery after surgery (ERAS) Pathway

**Fig. 2:**
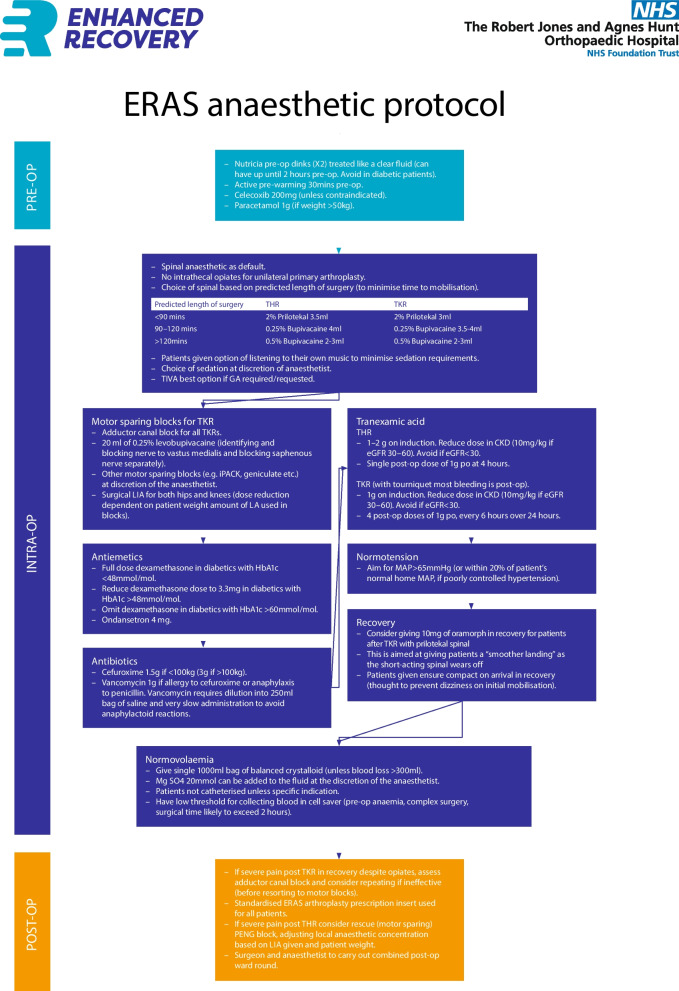
Anaesthetic Protocol for the enhanced recovery after surgery (ERAS) Pathway

In our tertiary high-volume orthopaedic centre, we developed a universal standardized non-selective ERAS pathway for all patients admitted for primary hip or knee arthroplasty, regardless of comorbidity status or age. Key components of this pathway include: pre-operative patient education through the “Joint School” service, nutritional optimization with high-energy drinks before and after surgery, standardized anaesthetic protocol and peri-operative analgesia, early physiotherapy to promote post-operative mobilization, and early telephone follow-up after discharge. Figure 1 illustrates the primary components of the implemented ERAS pathway. The joint school service is led by an arthroplasty nurse and provides patients with information about the procedure, expectations, and recovery period prior to the surgery date. The anaesthetic protocol is outlined in Fig. 2. Patients receive an X-ray following the procedure, which is reviewed by the operating surgeon. Physiotherapy rehabilitation begins after the X-ray, in the ward. Simple analgesia, such as paracetamol and codeine, is administered as required. Patients are discharged once they are medically stable and have met functional recovery goals, including safe independent ambulation (with appropriate aids), ability to perform basic self-care (transfers, toileting, stairs if applicable), tolerance of oral intake, adequate pain control, and understanding of prescribed medications and wound care. These criteria were essentially similar to those previously used in the standard care pathway. Telephone consultations are conducted with all patients on days 1 and 3 following surgery.

All patients who underwent elective primary hip or elective primary knee arthroplasty under ERAS from April 2023 to March 2024 were compared with a matched cohort from January 2018 to December 2019. This comparison period was chosen to avoid bias from the impact of the COVID-19 pandemic on patients’ length of stay, morbidity, and mortality during 2020–2022. Patients undergoing revision arthroplasty or hip arthroplasty for neck of femur fracture (NOF) were excluded.

A retrospective review of prospectively collected data was conducted, and patient-related data were collected using the electronic healthcare records. This study was exempt from IRB approval because all data were routinely collected for clinical and audit purposes.

1,818 and 4,968 patients underwent treatment within the ERAS and standard care groups, respectively. Patients were propensity-score matched at a 2:1 ratio based on procedure, age, sex, ASA grade, and BMI. Seven and 1,419 patients were subsequently excluded from the ERAS and standard care groups, respectively, resulting in 1,811 patients in the ERAS group and 3,549 patients in the standard care group for the final analysis.

An assessment of compliance with the key ERAS themes of pre-operative patient education, following defined peri-operative anaesthetic protocols, and early post-operative mobilisation with physiotherapy was conducted. Clinical outcomes included length of stay (LOS), 30-day readmission rates, overall infection rates, superficial infection rates, deep infection rates, and 30- and 90-day mortality rates.

Superficial Surgical Site Infection (SSI) was defined as an infection related to the surgical procedure that was treated with antibiotics without the need for further surgical intervention. Deep infection was defined as an infection related to the surgical procedure that required surgical washout, Debridement Antibiotic and Implant Retention (DAIR), or revision surgery. Infection rates were reported by the local infection control team in our centre and cross-checked with the UK Health Security Agency’s infection reports for our institution [13]. A composite postoperative complications outcome was defined as the occurrence of any infection (deep or superficial), hospital readmission, or 90-day mortality, with each patient counted once regardless of the number of events.

To assess whether observed changes in LOS could be explained by secular trends rather than ERAS implementation alone, an interrupted time series (ITS) analysis was performed. Monthly aggregated LOS was analysed using a stable pre-ERAS period (January 2018–December 2019) and the ERAS period (April 2023–March 2024). The intervening COVID-19 period (2020–2022) was excluded a priori due to substantial disruption to elective surgical pathways. Monthly mean LOS was used for the primary ITS analysis to allow estimation of level and trend changes with associated confidence intervals. Segmented linear regression models were fitted, including terms for baseline level, baseline trend, an indicator for ERAS implementation (level change), and a post-implementation trend change. To allow comparison across non-contiguous time periods, time was indexed sequentially. Heteroskedasticity- and autocorrelation-consistent (Newey–West) standard errors were used to account for serial correlation in monthly observations.

Subgroup analyses were conducted to compare length of stay and outcomes within five specific patient groups: (1) patients with primary hip arthroplasties, (2) patients with primary knee arthroplasties, (3) patients aged ≥ 80 years, (4) patients with ASA grades 3 and 4, and (5) patients with a BMI ≥ 40.

### Statistical analysis

Statistical analysis was conducted using SPSS (IBM Corp. Released 2023. IBM SPSS Statistics for Macintosh, Version 29.0.2.0. Armonk, NY: IBM Corp) and R software (R Core Team, 2020). Continuous data are summarized as mean ± standard deviation (SD) if normally distributed, and median ± interquartile range (IQR) if not normally distributed. Normality was tested using frequency histograms and Quantile–Quantile (Q-Q) plots. Categorical data are summarized as rates and frequencies.

Propensity score matching was performed using R software (R Core Team, 2020). The matching ratio was 2:1 (Standard Care: ERAS) with a caliper of 0.2. Length of stay between groups was compared using the Mann–Whitney U test (Wilcoxon Rank Sum test). The difference in the categorical outcomes between groups was assessed using the chi-square test.

## Results

Individuals ranged from 14–98 years in the standard care group (mean 67.36 ± 10.99 years) and 19–93 years in the ERAS group (mean 67.65 ± 10.78 years). The mean BMI was 30.48 ± 5.67 in standard care and 30.65 ± 5.42 in ERAS. ASA grade distribution was as follows: ASA 1 in 13.9% of standard care and 11.1% of ERAS; ASA 2 in 66.92% of standard care and 69.85% of ERAS; ASA 3 in 19.64% of standard care and 18.88% of ERAS; ASA 4 in 0.25% of standard care and 0.17% of ERAS. Patient demographics and baseline characteristics in the ERAS and Standard Care groups are presented in Table 1. No statistically significant differences were identified within any demographic variables between groups, indicating successful matching.

**Table 1:** Patient demographics and baseline characteristics in the ERAS and Standard Care groups

	**Standard Care (*n* = 3549)**	**ERAS (*n* = 1811)**	***P*-value**	**Matched SMD**	**Pre-matching SMD**
Age (mean ± SD) (years)	67.36 ± 10.99	67.64 ± 10.78	0.389	0.025	− 0.095
Sex (%)	Female: 51.8%	Female: 49.5%	0.111	0.047	− 0.131
	Male: 48.2%	Male: 50.5%			
BMI (mean ± SD)	30.48 ± 5.67	30.65 ± 5.42	0.307	0.030	0.196
ASA (mean ± SD)	2.07 ± 0.58	2.08 ± 0.55	0.480	0.021	0.064
Procedure (%)	Primary Hip: 45.8%	Primary Hip: 46.8%	0.129	0.039	− 0.001
	Primary Knee: 51.3%	Primary Knee: 51.3%		0.000	0.091
	Uni Knee: 2.9%	Uni Knee: 1.9%		0.039	− 0.227

In the ERAS group, 91.0% of cases followed the peri-operative anaesthetic protocol, and 50.8% attended the Joint School pre-operatively. The mode post-operative day of mobilisation was 0 (day 0: 70.2%, day 1: 29.2%, day ≥ 2: 0.6%).

The median length of stay (LOS) was 1 day (IQR 1–2) in the ERAS group and 3 days (IQR 2–4) in the Standard Care group (W = 5,415,769, *P* < 0.001). 30-day readmission rates were 1.7% in the ERAS group versus 2.1% in the Standard Care group (*P* = 0.41). The overall infection rate was 0.66% in the ERAS group compared to 1.15% in the Standard Care group (*P* = 0.11). The deep infection rate was 0.39% in the ERAS group versus 0.68% in the Standard Care group (*P* = 0.26). The superficial infection rate was 0.28% in the ERAS group versus 0.48% in the Standard Care group (*P* = 0.38). 30-day mortality rate was 0.11% in the ERAS group compared to 0.20% in the Standard Care group (*P* = 0.70). The 90-day mortality rate was 0.22% in the ERAS group versus 0.37% in the Standard Care group (*P* = 0.52). The composite postoperative complication rate (any infection, readmission, or 90-day mortality) was 2.0% in the ERAS group compared with 2.5% in the Standard Care group (*P* = 0.21). A summary of clinical outcomes comparing the ERAS and Standard Care groups has been presented in Table 2. Table 3 demonstrates the LOS between standard care and ERAS, stratified by age category. Table 4 demonstrates the LOS between standard care and ERAS, stratified by ASA grade.

**Table 2:** Summary of perioperative outcomes between Standard Care and ERAS

Entire Cohort					
**Outcome**	**ERAS n/N (%) [95% CI]**	**Standard Care n/N (%) [95% CI]**	**Abs risk diff (pp) [95% CI]**	**RR (95% CI)**	***P*-value**
Readmission	31/1811 (1.71%)	74/3549 (2.09%)	− 0.37 [− 1.10 to 0.45]	0.82 (0.54–1.24)	0.41#
Any infection	12/1811 (0.66%)	41/3549 (1.16%)	− 0.49 [− 0.99 to 0.08]	0.57 (0.30–1.09)	0.11#
Deep infection	7/1811 (0.39%)	24/3549 (0.68%)	− 0.29 [− 0.67 to 0.18]	0.57 (0.25–1.32)	0.26#
Superficial infection	5/1811 (0.28%)	17/3549 (0.48%)	− 0.20 [− 0.53 to 0.21]	0.58 (0.21–1.56)	0.38#
30-day mortality	2/1811 (0.11%)	7/3549 (0.20%)	− 0.09 [− 0.31 to 0.22]	0.56 (0.12–2.69)	0.70#
90-day mortality	4/1811 (0.22%)	13/3549 (0.37%)	− 0.15 [− 0.44 to 0.23]	0.60 (0.20–1.85)	0.52#
Composite outcome	36/1811 (1.99%)	90/3549 (2.54%)	− 0.55 [− 1.34 to 0.34]	0.78 (0.53–1.15)	0.21#

n = number of events; N = total number of patients in the group; Abs = Absolute; diff = difference; RR = relative risk; CI = confidence interval

^#^: Chi^2^

**Table 3:** Length of Stay between Standard Care and ERAS stratified by age category

	**Age (years)**	**Frequency**	**%**	**Mean**	**Median**	**SD**
Standard Care	< 40	39	1.1%	3.62	3	1.74
	40–60	886	24.96%	3.31	3	6.55
	60–80	2251	63.43%	3.77	3	3.89
	> 80	373	10.51%	5.74	4	4.74
ERAS	< 40	20	1.1%	0.6	1	0.5
	40–60	416	22.97%	1.37	1	1.48
	60–80	1205	66.54%	1.77	1	2.49
	> 80	170	9.39%	2.95	2	3.4

**Table 4:** Length of Stay between Standard Care and ERAS stratified by ASA grade

	**ASA**	**Frequency**	**%**	**Mean**	**Median**	**SD**
Standard Care	1	468	13.19%	2.87	3	1.42
	2	2375	66.92%	3.58	3	3.79
	3	697	19.64%	5.46	4	7.99
	4	9	0.25%	5.56	5	2.7
ERAS	1	201	11.1%	1.18	1	0.88
	2	1265	69.85%	1.5	1	1.53
	3	342	18.88%	3.15	2	4.44
	4	3	0.17%	1.67	2	0.58

**Fig. 3:**
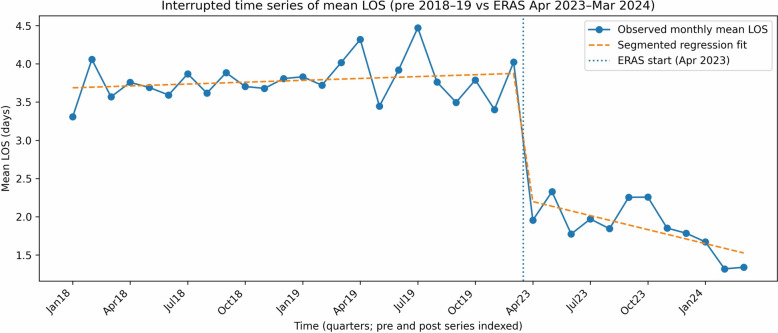
Interrupted time series (ITS) analysis of monthly mean length of stay (LOS) before and after implementation of an enhanced recovery after surgery (ERAS) pathway

In ITS analysis of monthly mean LOS, ERAS implementation was associated with a significant immediate reduction in LOS of 1.6 days (95% CI: − 2.0 to − 1.3; *P* < 0.001). There was no significant pre-ERAS temporal trend in LOS (+ 0.01 days per month; *P* = 0.27). Following ERAS implementation, there was evidence of a further reduction in LOS over time, with a post-implementation trend change of − 0.07 days per month (95% CI: − 0.11 to − 0.03; *P* = 0.002). (Fig. 3) In sensitivity analysis using monthly median LOS, median LOS decreased from 3 days pre-ERAS to 1 day post-ERAS, consistent with the mean-based analysis.

Subgroup analysis of patients who had hip arthroplasties and knee arthroplasties (Table 5) shows a consistent significant difference in the length of stay of 2 days between ERAS and standard care, with non-statistically significant differences in the perioperative outcomes.

**Table 5:** Summary of Perioperative Outcomes for Hip and Knee Arthroplasties

Outcome	Standard Care Hip (n = 1624)	ERAS Hip (n = 847)	p-value (Hip)	Standard Care Knee (n = 1925)	ERAS Knee (n = 964)	*P*-value (Knee)
Length of Stay (Median, IQR)	3 (2–4)	1 (1–2)	< 0.001*	3 (2–4)	1 (1–2)	< 0.001*
Readmission (%)	33 (2.03%)	9 (1.06%)	0.11#	41 (2.13%)	22 (2.28%)	0.90#
Infection (%)	19 (1.17%)	3 (0.35%)	0.07#	22 (1.14%)	9 (0.93%)	0.75#
Superficial Infection (%)	8 (0.49%)	2 (0.24%)	0.54#	9 (0.47%)	3 (0.31%)	0.76#
Deep Infection (%)	11 (0.68%)	1 (0.12%)	0.11#	13 (0.68%)	6 (0.62%)	1.00#
30-Day Mortality (%)	4 (0.25%)	0 (0.00%)	0.36#	3 (0.16%)	2 (0.21%)	1.00#
90-Day Mortality (%)	8 (0.49%)	1 (0.12%)	0.26#	5 (0.26%)	3 (0.31%)	1.00#

**Length of Stay in reported in days**

^*^: Wilcoxon Rank Test; #: Chi^2^

Subgroup analysis of patients aged ≥ 80 years included 215 patients in the ERAS group versus 437 in the Standard Care group. The median LOS was 2 days in the ERAS group (IQR 1–3) compared to 5 days in the Standard Care group (IQR 4–8) (*P* < 0.001). The median difference in LOS between the two groups was 3 days, compared to a 2-day difference in the entire cohort. Table 6 presents the difference in perioperative outcomes between ERAS and standard care in patients aged ≥ 80 years.

**Table 6:** Perioperative outcomes between ERAS and standard care in patients aged ≥ 80 years

Age ≥ 80 years			
Outcome	**Standard Care (n = 437)**	**ERAS (n = 215)**	***P*-value**
Length of Stay (Median (IQR))	5 (4–8)	2 (1–3)	< 0.001*
Readmission (%)	12 (2.7)	3 (1.4)	0.422#
Infection (%)	7 (1.6)	1 (0.5)	0.389#
Superficial Infection (%)	3 (0.7)	0 (0.0)	0.547#
Deep Infection (%)	4 (0.9)	1 (0.5)	0.887#
30 Day Mortality (%)	1 (0.2)	0 (0.0)	1.000#
90 Day Mortality (%)	4 (0.9)	0 (0.0)	0.382#

**Length of Stay is reported in days**

^*^: Wilcoxon Rank Test; #: Chi^2^

Subgroup analysis for patients with ASA grades 3 and 4 included 345 patients in the ERAS group versus 706 in the Standard Care group. The median LOS was 2 days in the ERAS group (IQR 1–3) compared to 4 days in the Standard Care group (IQR 3–6) (*P* < 0.001). Table 7 presents the difference in perioperative outcomes between ERAS and standard care in patients with ASA 3 and 4.

**Table 7:** Perioperative outcomes between ERAS and standard care in patients with ASA 3 and 4

ASA 3 and 4			
Outcome	**Standard Care (n = 706)**	**ERAS (n = 345)**	***P*-value**
Length of Stay (Median (IQR))	4 (3–6)	2 (1–3)	< 0.001*
Readmission (%)	24 (3.4)	7 (2.0)	0.298#
Infection (%)	14 (1.9)	4 (1.1)	0.475#
Superficial Infection (%)	6 (0.8)	1 (0.3)	0.519#
Deep Infection (%)	8 (1.1)	3 (0.9)	0.943#
30 Day Mortality (%)	7 (1.0)	3 (0.9)	0.395#
90 Day Mortality (%)	12 (1.7)	4 (1.1)	0.229#

**Length of Stay is reported in days**

^*^: Wilcoxon Rank Test; #: Chi^2^

Subgroup analysis for patients with a BMI ≥ 40 included 105 patients in the ERAS group versus 192 in the Standard Care group. The median LOS was 1 day in the ERAS group (IQR 1–3) compared to 3 days in the Standard Care group (IQR 2–5). A LOS difference of 2 days aligns with the results observed in the entire cohort. Table 8 presents the difference in perioperative outcomes between ERAS and standard care in patients with a BMI ≥ 40.

**Table 8:** perioperative outcomes between ERAS and standard care, in patients with BMI ≥ 40

BMI ≥ 40			
Outcome	**Standard Care (n = 192)**	**ERAS (n = 105)**	***P*-value**
Length of Stay (Median (IQR))	3 (2–5)	1 (1–3)	< 0.001*
Readmission (%)	8 (4.1)	2 (1.9)	0.486#
Infection (%)	4 (2.1)	2 (1.9)	1.000#
Superficial Infection (%)	2 (1.0)	0 (0.0)	0.758#
Deep Infection (%)	2 (1.0)	2 (1.9)	0.928#
30 Day Mortality (%)	0 (0.0)	0 (0.0)	1.000#
90 Day Mortality (%)	1 (0.5)	0 (0.0)	1.000#

**Length of Stay is reported in days**

^*^: Wilcoxon Rank Test; #: Chi^2^

The rates of 30-day readmission, overall infection, superficial infection, deep infection, or 30- and 90-day mortality were not statistically significant between the ERAS and standard care groups across all three subgroup analyses, consistent with the results for the entire cohort.

## Discussion

A standardized ERAS service has been set up in a high-volume tertiary orthopaedic centre with high compliance rates to defined anaesthetic protocol and early post-operative physiotherapy rehabilitation. Non-selective ERAS reduced LOS for primary hip and knee arthroplasty patients with ASA grades I through IV, with a statistically significant median difference of 2 days. The 30-day readmission, infection, and 30- and 90-day mortality rates were lower in the ERAS group, though these differences did not reach statistical significance.

Previous studies have outlined the efficacy of ERAS in reducing patients’ LOS; however, these were patient-selected and mostly included those with ASA I, II, or younger age groups [10–12, 14, 15]. This study demonstrates that the efficacy of ERAS extends to the morbid and elderly patients. It also confirms the safety in implementing a non-selective ERAS pathway across all patients undergoing primary hip and knee arthroplasty, regardless of age or comorbid status.

This reduction in LOS for hip and knee arthroplasty patients would have significant benefits, including a reduction in healthcare system expenditure. The cost-effectiveness of patient-selected ERAS pathways has been emphasized by several studies [3, 6, 16–18]. Milligan et al. reported a £757.26 (95% CI £−1,200.96 to £−313.56) cost difference per patient in their pilot study comparing a matched group of 200 patients between standard care and ERAS [18]. In our study, a focused cost analysis was performed from the NHS provider perspective, estimating cost differences attributable solely to changes in length of stay during the index admission. Rather than a full economic evaluation, we conducted an LOS-based inpatient cost analysis comparing non-selective ERAS with standard care using a matched sample of 1,811 patients per group. The cost of a general ward bed per day is £345 according to the NHS England National Cost Collection (2020/21 price base) [19]. The mean length of stay was 1.78 days (95% CI 1.67, 1.89) in the ERAS group versus 3.86 days (95% CI 3.64, 4.08) in the standard care group. The resulting LOS-attributable difference in cost per patient was £718.60 (95% CI £602.56–£832.64), corresponding to a total estimated reduction in average inpatient bed-day costs of £1.3 million (95% CI £1.1–£1.5 million) at our institution over the ERAS implementation period (April 2023–March 2024), which included 1,818 patients. These estimates reflect average rather than marginal costs and do not account for costs related to readmissions or complications. Other indirect cost reductions could include staff reduction and maximizing operating theatre utilization due to quicker turnover from freeing up hospital beds more rapidly.

Subgroup analysis highlighted a greater reduction in LOS in the ≥ 80 years old group. The benefits of implementing ERAS could therefore extend beyond cost reduction, particularly for elderly patients with multiple comorbidities [20]. These patients are at risk of developing medical complications such as chest infections and venous thromboembolism as a consequence of prolonged hospital stays [20]. Encouraging early mobilization and expediting rehabilitation for this group could further reduce medical complications [6, 14, 16, 21]. However, it is important to highlight that this nonselective ERAS pathway does not force early discharge; patients are only discharged once all the discharge criteria are met [22]. This approach is more applicable in a broader healthcare context compared to day-case arthroplasty units, which do not have the option for inpatient stay following surgery due to bed and staff unavailability. Conversely, Liu et al. reported increased cardiopulmonary complications following discharge on day 0for TKA and THA patients compared to discharge on day 1 [23]. This emphasises the importance of avoiding forced discharge and highlights that an expedited patient-tailored approach is likely safer.

Compliance with the ERAS protocol was associated with a greater reduction in length of stay. This is consistent with the findings of Ripollés-Melchor et al. in the POWER2 study, which showed that adherence to the ERAS protocol was associated with reduced LOS, fewer complications, and improved outcomes. [11] However, they noted that these benefits were primarily observed in younger and healthier patients [11]. Similarly, Morrell et al., in their systematic review comparing ERAS to standard care, reported analogous findings. [10] The heterogeneity of their data due to the involvement of multiple centres with different ERAS protocols may, however, undermine these results. In contrast, this study challenges the finding of a better impact of ERAS on younger, healthier patients. We believe this could pave the way for broader implementation of such pathways and expand inclusion to involve revision cases.

We acknowledge that the retrospective and observational nature of this study is a limitation. Furthermore, the analysis did not include social or racial characteristics of the patients, which may have influenced the outcomes. Although mortality differences between the groups were not statistically significant, the analysis was likely underpowered to detect such differences.

Further research is recommended to investigate the long-term effects of ERAS on one-year patient-reported outcome measures (PROMs) compared with the standard care cohort. Moreover, patient-specific factors, anaesthetic variables, and postoperative rehabilitation elements associated with the success or failure of early discharge should be evaluated to improve the optimisation of these pathways.

In conclusion, non-selective ERAS was safe and effective in reducing LOS for patients undergoing primary THA and TKA across all age groups and varying comorbidity statuses. Although perioperative morbidity and mortality were less in ERAS, these changes did not reach statistical significance.

## Data Availability

The anonymised datasets used for the current study is available upon request by the editor or reviewers.
